# A novel model for predicting prolonged stay of patients with type-2 diabetes mellitus: a 13-year (2010–2022) multicenter retrospective case–control study

**DOI:** 10.1186/s12967-023-03959-1

**Published:** 2023-02-07

**Authors:** Juntao Tan, Zhengyu Zhang, Yuxin He, Yue Yu, Jing Zheng, Yunyu Liu, Jun Gong, Jianjun Li, Xin Wu, Shengying Zhang, Xiantian Lin, Yuxi Zhao, Xiaoxin Wu, Songjia Tang, Jingjing Chen, Wenlong Zhao

**Affiliations:** 1 Operation Management Office, Affiliated Banan Hospital of Chongqing Medical University, Chongqing, 401320 China; 2 Medical Records Department, The First Affiliated Hospital, Zhejiang University School of Medicine, Hangzhou, 310003 Zhejiang China; 3 Department of Medical Administration, Affiliated Banan Hospital of Chongqing Medical University, Chongqing, 401320 China; 4 Senior Bioinformatician Department of Quantitative Health Sciences Mayo Clinic, Rochester, MN 55905 USA; 5 Medical Records Department, The Second Affiliated Hospital of Chongqing Medical University, Chongqing, 400010 China; 6 Department of Information Center, The University Town Hospital of Chongqing Medical University, Chongqing, 401331 China; 7 Department of Cardiothoracic Surgery, Affiliated Banan Hospital of Chongqing Medical University, Chongqing, 401320 China; 8 Department of Gastrointestinal Surgery, The Third People’s Hospital of Chongqing, Chongqing Medical University, Chongqing, 400038 China; 9 Department of Respiratory, Yinzhou Second Hospital, Ningbo, 315153 Zhejiang China; 10 State Key Laboratory for Diagnosis and Treatment of Infectious Diseases, National Clinical Research Centre for Infectious Diseases, The First Affiliated Hospital, Zhejiang University School of Medicine, 79 Qing Chun Road, Hangzhou, 310003 Zhejiang China; 11 Plastic and Aesthetic Surgery Department, Affiliated Hangzhou First People’s Hospital, Zhejiang University School of Medicine, Hangzhou, 310000 Zhejiang China; 12 Department of Digital Urban Governance, Zhejiang University City College, Hangzhou, 310015 Zhejiang China; 13 College of Medical Informatics, Chongqing Medical University, Chongqing, 400016 China; 14 Medical Data Science Academy, Chongqing Medical University, Chongqing, 400016 China

**Keywords:** Type-2 diabetes mellitus, Prolonged stay, Prediction model, Nomogram, Online service

## Abstract

**Background:**

Length of stay (LOS) is an important metric for evaluating the management of inpatients. This study aimed to explore the factors impacting the LOS of inpatients with type-2 diabetes mellitus (T2DM) and develop a predictive model for the early identification of inpatients with prolonged LOS.

**Methods:**

A 13-year multicenter retrospective study was conducted on 83,776 patients with T2DM to develop and validate a clinical predictive tool for prolonged LOS. Least absolute shrinkage and selection operator regression model and multivariable logistic regression analysis were adopted to build the risk model for prolonged LOS, and a nomogram was taken to visualize the model. Furthermore, receiver operating characteristic curves, calibration curves, and decision curve analysis and clinical impact curves were used to respectively validate the discrimination, calibration, and clinical applicability of the model.

**Results:**

The result showed that age, cerebral infarction, antihypertensive drug use, antiplatelet and anticoagulant use, past surgical history, past medical history, smoking, drinking, and neutrophil percentage-to-albumin ratio were closely related to the prolonged LOS. Area under the curve values of the nomogram in the training, internal validation, external validation set 1, and external validation set 2 were 0.803 (95% CI [confidence interval] 0.799–0.808), 0.794 (95% CI 0.788–0.800), 0.754 (95% CI 0.739–0.770), and 0.743 (95% CI 0.722–0.763), respectively. The calibration curves indicated that the nomogram had a strong calibration. Besides, decision curve analysis, and clinical impact curves exhibited that the nomogram had favorable clinical practical value. Besides, an online interface (https://cytjt007.shinyapps.io/prolonged_los/) was developed to provide convenient access for users.

**Conclusion:**

In sum, the proposed model could predict the possible prolonged LOS of inpatients with T2DM and help the clinicians to improve efficiency in bed management.

**Supplementary Information:**

The online version contains supplementary material available at 10.1186/s12967-023-03959-1.

## Introduction

Diabetes mellitus, a common metabolic disease characterized by chronic hyperglycemia, endangers human health [1, 2]. The 10th edition of the Diabetes Map reported that approximately 537 million adults worldwide would suffer from diabetes by 2021. By 2030, this number is expected to rise to 643 million, and by 2045, it will further rise to 783 million. Currently, there are the largest number of people with diabetes in China, and the number is gradually climbing year by year [3]. Among all diabetes diseases, type-2 diabetes mellitus (T2DM) has the highest prevalence, accounting for more than 90% of all [4, 5].

T2DM is not only a serious threat to people's health but also leads to a sharp increase in medical expenses, causing a heavy economic burden to patients and society [6, 7]. According to a report released by the International Diabetes alliance, the global medical expenses for treating diabetic patients reached US $850 billion in 2017, accounting for 12.5% of the global health and medical expenses. Among them, the medical expenses for diabetes in China reached US $110 billion, ranking second in the world after the United States (US $348 billion) [3]. Prolonged LOS in hospitals has been identified as one of the main reasons for the increased expenses [8–10]. Previous studies have studied the factors associated with prolonged LOS for several common diseases [11–14], and risk factors such as age, nutritional status, body mass index, underlying diseases (e.g. hypertension, arrhythmias, and chronic pulmonary disease), and common laboratory measures (e.g. prothrombin time, neutrophils, and ejection fraction) have been reported. However, there are few studies on prolonged LOS of inpatients with T2DM, and the relevant studies had used only a small number of participants and did not evaluate a wide variety of clinical factors.

Therefore, in this study, we recruited a large, multicenter patient population with T2DM. We aimed to: (i) identifying novel risk predictors of prolonged LOS of patients with T2DM, with the hope of remediating and preventing unwanted prolonged LOS, and (ii) constructing an online predictive service to provide clinicians with a simple and precise personalized prediction function of LOS of inpatients.

## Methods

### Study design and patients

In this study, the clinical data containing 120,073 patients with T2DM were collected from six hospitals in China, of which 83,776 patients passed quality control for the final analysis, according to the Transparent Reporting of a Multivariable Prediction Model for Individual Prognosis or Diagnosis guideline [15]. A total of 2224 patients were recruited from Yongchuan Hospital of Chongqing Medical University; 34,888 from the Second Affiliated Hospital of Chongqing Medical University, 14,767 from the University Town Hospital of Chongqing Medical University, and 20,100 from the Third Affiliated Hospital of Chongqing Medical University. Patients from these four centers were divided randomly (7:3) into training (n = 50,385) and internal validation (n = 21,594) sets. Patients recruited from Chongqing Southeast Hospital (n = 7018) and People's Hospital of Tongjiang District (n = 4779) were used as external validation set 1 and external validation set 2, respectively. Additional file 1: Table S1. displays the data from the six hospitals.

This study was approved by the Ethics Committee of the Affiliated Banan Hospital of Chongqing Medical University and was conducted in accordance with the Declaration of Helsinki and Good Clinical Practice guidelines. Informed consent for participation was not required for this study owing to its retrospective design, and the study was conducted in accordance with the national legislation and institutional requirements.

### Inclusion and exclusion criteria

The inclusion criteria includes: (i) data obtained from 2010 to 2022, and (ii) hospitalization(s) for T2DM. The exclusion criteria includes: (i) age < 18 years, (ii) death during hospitalization, (iii) discharge against medical advice, and (iv) patients with > 30% missing data. The selection process is illustrated in Additional file 1: Fig. S1.

### Data collection

A total of 31 candidate variables were selected to identify a prolonged LOS. Specifically, we explored age, sex, insurance type, age-adjusted charlson comorbidity index score [16], hypertension, coronary heart disease, cerebral infarction, hyperlipidemia, antihypertensive drug use, statin use, antiplatelet and anticoagulant use, past surgical history (PSH), past medical history(PMH), smoking, drinking, systolic and diastolic blood pressure, pulse, aspartate aminotransferase, alanine aminotransferase, triglycerides, neutrophil-to-lymphocyte ratio, platelet-to-lymphocyte ratio, lymphocyte-to-monocyte ratio, neutrophil percentage-to-albumin ratio (NPAR), creatinine, uric acid, low- and high-density lipoprotein cholesterol, fasting glucose, and glomerular filtration rate. All biomarkers only retain the values measured for the first time after admission.

### Definition

LOS was calculated from the date of admission to the date of checkout. A LOS exceeding the third quartile value of the study population was defined as prolonged LOS [17–22]. Specifically, hospitalization < 13 days was defined as normal LOS; whereas, hospitalization ≥ 14 days was defined as prolonged LOS.

The type of insurance chosen by individuals reflects the economic burden borne by the individuals and families. In this study, insurance type can be grouped to: urban employee medical insurance, urban resident medical insurance, other insurance, and full self-payment [23].

### Statistical analyses

Statistical analyses were performed using SPSS 22.0 and R (version 4.0.2, Vienna, Austria). Least absolute shrinkage and selection operator regression model and multivariable logistic regression analysis were used to identify the independent factors [24]. A nomogram was constructed based on these independent factors, and area under the receiver operating characteristic curve was used to evaluate the discriminative ability of the nomogram [25]. Calibration curves were used to evaluate calibration of the nomogram [26]. Furthermore, decision curve analysis and clinical impact curve were used to demonstrate the clinical applicability of the nomogram [27–29]. The multiple imputation method was used to fill in missing continuous variables [30, 31]. In each simulation dataset, missing data will be filled by Monte Carlo method. At this point, the standard statistical method can be applied to each simulated dataset, and the estimated results and the confidence interval when missing values are introduced are given by combining the output results. The enumerative data were expressed as rates and percentages, and the chi-square test was used for comparisons between groups. The quantitative variables did not show a normal distribution and were represented by median and interquartile range [M(Q25–Q75)]. Comparisons between groups were performed using the Mann–Whitney *U* test. All statistical analyses were two-sided, and statistical significance was set at *P* < 0.05.

## Results

### Subject characteristics

The clinical characteristics of the subjects in the training and internal validation sets were summarized in Table 1. No significant difference was found between the training and internal validation sets in terms of age, sex, insurance type, hypertension, coronary heart disease, cerebral infarction, hyperlipidemia, statin use, antiplatelet and anticoagulant use, PSH, PMH, smoking, drinking, or all laboratory variables (*P* > 0.05).

**Table 1:** Demographic and clinical characteristics of the training and internal validation sets

Variables	Total (N = 71,979)	Training set (N = 50,385)	Internal validation set (N = 21,594)	*P* values
Age (IQR, year)	63.00(51.00,72.00)	63.00(51.00,72.00)	63.00(51.00,72.00)	0.695
Sex (n, %)				0.117
Female	39,051(54.25)	27,432(54.44)	11,619(53.81)	
Male	32,928(45.75)	22,953(45.56)	9975(46.19)	
Insurance type (n, %)				0.567
Full self-pay	6798(9.44)	4797(9.52)	2001(9.27)	
UEMI	16,887(23.46)	11,859(23.54)	5028(23.28)	
URMI	45,347(63.00)	31,671(62.86)	13,676(63.33)	
Other insurance	2947(4.10)	2058(4.08)	889(4.12)	
ACCI score (n, %)				0.020
0–4	28,730(39.92)	20,111(39.92)	8619(39.91)	
5–7	30,305(42.10)	21,335(42.34)	8970(41.54)	
> 7	12,944(17.98)	8939(17.74)	4005(18.55)	
Hypertension (n, %)				0.532
Yes	34,910(48.50)	24,398(48.42)	10,512(48.68)	
No	37,069(51.50)	25,987(51.58)	11,082(51.32)	
CHD (n, %)				0.273
Yes	14,896(20.69)	10,372(20.59)	4524(20.95)	
No	57,083(79.31)	40,013(79.41)	17,070(79.05)	
CI (n, %)				0.461
Yes	8514(11.83)	5930(11.77)	2584(11.97)	
No	63,465(88.17)	44,455(88.23)	19,010(88.03)	
Hyperlipidemia (n, %)				0.901
Yes	9914(13.77)	6934(13.76)	2980(13.80)	
No	62,065(86.23)	43,451(86.24)	18,614(86.20)	
Antihypertensive drug use (n, %)				0.003
Yes	31,618(43.93)	21,950(43.56)	9668(44.77)	
No	40,361(56.07)	28,435(56.44)	11,926(55.23)	
Statins use (n, %)				0.057
Yes	26,279(36.51)	18,282(36.28)	7997(37.03)	
No	45,700(63.49)	32,103(63.72)	13,597(62.97)	
Antiplatelet and anticoagulant use (n, %)				0.051
Yes	28,920(40.18)	20,126(39.94)	8794(40.72)	
No	43,059(59.82)	30,259(60.06)	12,800(59.28)	
PSH (n, %)				0.530
Yes	37,366(51.91)	26,117(51.83)	11,249(52.09)	
No	34,613(48.09)	24,268(48.17)	10,345(47.91)	
PMH (n, %)				0.671
Yes	57,451(79.82)	40,194(79.77)	17,257(79.92)	
No	14,528(20.18)	10,191(20.23)	4337(20.08)	
Smoking history (n, %)				0.264
Yes	21,492(29.86)	14,981(29.73)	6511(30.15)	
No	50,487(70.14)	35,404(70.27)	15,083(69.85)	
Drinking history (n, %)				0.401
Yes	18,225(25.32)	12,712(25.23)	5513(25.53)	
No	53,754(74.68)	37,673(74.77)	16,081(74.47)	
SBP (IQR, mmHg)	132.00(120.00,146.00)	132.00(120.00,146.00)	131.00(120.00,146.00)	0.262
DBP (IQR, mmHg)	78.00(70.00,86.00)	78.00(70.00,86.00)	78.00(70.00,86.00)	0.387
Pulse (IQR, bpm)	80.00(74.00,90.00)	80.00(75.00,90.00)	80.00(74.00,90.00)	0.716
AST (IQR, IU/L)	20.00(16.00,26.20)	20.00(16.00,26.30)	20.00(16.00,26.10)	0.899
ALT (IQR, IU/L)	19.00(13.00,29.00)	19.00(13.00,29.00)	19.00(13.00,29.00)	0.836
TGs (IQR, mmol/l)	1.56(1.10,2.34)	1.57(1.10,2.344)	1.55(1.10,2.33)	0.135
NLR (IQR)	2.95(1.99,4.69)	2.95(1.99,4.67)	2.97(1.99,4.72)	0.627
PLR (IQR)	121.15(90.27,166.06)	121.12(90.23,165.96)	121.28(90.32,166.35)	0.736
LMR (IQR)	3.81(2.57,5.42)	3.82(2.58,5.43)	3.79(2.56,5.39)	0.085
NPAR (IQR, ml/g)	16.74(14.26,20.00)	16.73(14.25,20.00)	16.75(14.29,20.00)	0.523
CREA (IQR, umol/l)	64.60(52.10,81.90)	64.60(52.10,81.80)	64.64(52.20,81.99)	0.393
UA (IQR, umol/l)	324.46(263.70,396.40)	325.00(263.30,396.60)	323.22(264.51,395.90)	0.442
LDL-C (IQR, mmol/l)	2.45(1.86,3.07)	2.44(1.86,3.07)	2.45(1.85,3.07)	0.741
HDL-C (IQR, mmol/l)	1.10(0.92,1.33)	1.10(0.92,1.33)	1.10(0.92,1.33)	0.603
FG (IQR, mmol/l)	7.48(5.76,10.61)	7.48(5.76,10.60)	7.48(5.75,10.62)	0.870
GFR (IQR, ml/min)	97.99(76.59,120.14)	98.00(76.52,120.17)	97.95(76.79,119.99)	0.913

UEMI, urban employee medical insurance; URMI, urban resident medical insurance; ACCI, age-adjusted charlson comorbidity score; CHD, coronary heart disease; CI, cerebral infarction; PSH, past surgical history; PMH, past Medical History; SBP, systolic blood pressure; DBP, diastolic blood pressure; AST, aspartate aminotransferase; ALT, alanine aminotransferase; TGs, triglycerides; NLR, neutrophil-to-lymphocyte ratio;PLR, platelet-lymphocyte ratio; LMR, lymphocyte-to-monocyte ratio; NPAR, neutrophil percentage-to-albumin ratio; CREA, creatinine; UA, uric acid; LDL-C, low density lipoprotein cholesterol; HDL-C, high density lipoprotein cholesterol; FG, fasting glucose; GFR, glomerular filtration rate; bpm, beat per minute; IQR, interquartile range

### Selection of predictors

Patients from the training set were stratified into one of the two groups according to LOS: normal LOS (n = 38,026) and prolonged LOS (n = 12,359). Univariate analysis identified that the following variables were associated with prolonged LOS: age, sex, insurance type, age-adjusted charlson comorbidity index score, hypertension, coronary heart disease, cerebral infarction, antihypertensive drug use, statin use, antiplatelet and anticoagulant use, PSH, PMH, smoking, drinking, systolic and diastolic blood pressure, aspartate aminotransferase, triglycerides, neutrophil-to-lymphocyte ratio, platelet-to-lymphocyte ratio, lymphocyte-to-monocyte ratio, NPAR, creatinine, uric acid, low-density lipoprotein cholesterol, high-density lipoprotein cholesterol, fasting glucose, and glomerular filtration rate (Table 2).

**Table 2:** Demographic and clinical characteristics associated with a prolonged LOS as assessed in the training set

Variables	Normal LOS (N = 38,026)	Prolonged LOS (N = 12,359)	*P* values
Age (IQR, year)	61.00(48.00,71.00)	68.00(58.00,76.00)	< 0.001
Sex (n, %)			< 0.001
Female	21,566(56.71)	5866(47.46)	
Male	16,460(43.29)	6493(52.54)	
Insurance type (n, %)			< 0.001
Full self-pay	3931(10.34)	866(7.01)	
UEMI	9527(25.05)	2332(18.87)	
URMI	22,871(60.15)	8800(71.20)	
Other insurance	1697(4.46)	361(2.92)	
ACCI score (n, %)			< 0.001
0–4	16,936(44.54)	3175(25.69)	
5–7	15,420(40.55)	5915(47.86)	
> 7	5670(14.91)	3269(26.45)	
Hypertension (n, %)			< 0.001
Yes	16,736(44.01)	7662(62.00)	
No	21,290(55.99)	4697(38.00)	
CHD (n, %)			< 0.001
Yes	6768(17.8)	3604(29.16)	
No	31,258(82.2)	8755(70.84)	
CI (n, %)			< 0.001
Yes	3226(8.48)	2704(21.88)	
No	34,800(91.52)	9655(78.12)	
Hyperlipidemia (n, %)			0.123
Yes	5285(13.9)	1649(13.34)	
No	32,741(86.1)	10,710(86.66)	
Antihypertensive drug use (n, %)			< 0.001
Yes	13,379(35.18)	8571(69.35)	
No	24,647(64.82)	3788(30.65)	
Statins use (n, %)			< 0.001
Yes	12,485(32.83)	5797(46.91)	
No	25,541(67.17)	6562(53.09)	
Antiplatelet and anticoagulant use (n, %)			< 0.001
Yes	12,319(32.4)	7807(63.17)	
No	25,707(67.6)	4552(36.83)	
PSH (n, %)			< 0.001
Yes	17,730(46.63)	8387(67.86)	
No	20,296(53.37)	3972(32.14)	
PMH (n, %)			< 0.001
Yes	28,342(74.53)	11,852(95.90)	
No	9684(25.47)	507(4.10)	
Smoking history (n, %)			< 0.001
Yes	9672(25.44)	5309(42.96)	
No	28,354(74.56)	7050(57.04)	
Drinking history (n, %)			< 0.001
Yes	8047(21.16)	4665(37.75)	
No	29,979(78.84)	7694(62.25)	
SBP (IQR, mmHg)	130.00(120.00,145.00)	135.00(122.00,150.00)	< 0.001
DBP (IQR, mmHg)	79.00(71.00,86.00)	78.00(70.00,86.00)	0.003
Pulse (IQR, bpm)	80.00(75.00,90.00)	80.00(74.00,91.00)	0.442
AST (IQR, IU/L)	19.80(16.00,26.00)	20.00(16.00,28.00)	< 0.001
ALT (IQR, IU/L)	19.00(13.00,29.00)	19.00(13.00,30.00)	0.585
TGs (IQR, mmol/l)	1.61(1.13,2.42)	1.45(1.04,2.12)	< 0.001
NLR (IQR)	2.80(1.93,4.35)	3.48(2.26,5.94)	< 0.001
PLR (IQR)	117.93(88.79,158.78)	132.82(96.04,190.11)	< 0.001
LMR (IQR)	3.97(2.73,5.59)	3.31(2.17,4.86)	< 0.001
NPAR (IQR, ml/g)	16.30(14.01,19.47)	18.07(15.33,21.53)	< 0.001
CREA (IQR, umol/l)	62.80(50.90,78.50)	71.00(56.80,93.90)	< 0.001
UA (IQR, umol/l)	324.28(264.45,394.19)	327.62(259.00,404.57)	0.132
LDL-C (IQR, mmol/l)	2.48(1.90,3.10)	2.32(1.75,2.96)	< 0.001
HDL-C (IQR, mmol/l)	1.12(0.93,1.34)	1.06(0.88,1.29)	< 0.001
FG (IQR, mmol/l)	7.32(5.64,10.27)	8.09(6.14,11.54)	< 0.001
GFR (IQR, ml/min)	100.90(80.66,123.40)	89.01(63.55,109.29)	< 0.001

UEMI, urban employee medical insurance; URMI, urban resident medical insurance; ACCI, age-adjusted charlson comorbidity score; CHD, coronary heart disease; CI, cerebral infarction; PSH, past surgical history; PMH, past Medical History; SBP, systolic blood pressure; DBP, diastolic blood pressure; AST, aspartate aminotransferase; ALT, alanine aminotransferase; TGs, triglycerides; NLR, neutrophil-to-lymphocyte ratio;PLR, platelet-lymphocyte ratio; LMR, lymphocyte-to-monocyte ratio; NPAR, neutrophil percentage-to-albumin ratio; CREA, creatinine; UA, uric acid; LDL-C, low density lipoprotein cholesterol; HDL-C, high density lipoprotein cholesterol; FG, fasting glucose; GFR, glomerular filtration rate; bpm, beat per minute; LOS, length of stay; IQR, interquartile range

**Fig. 1:**
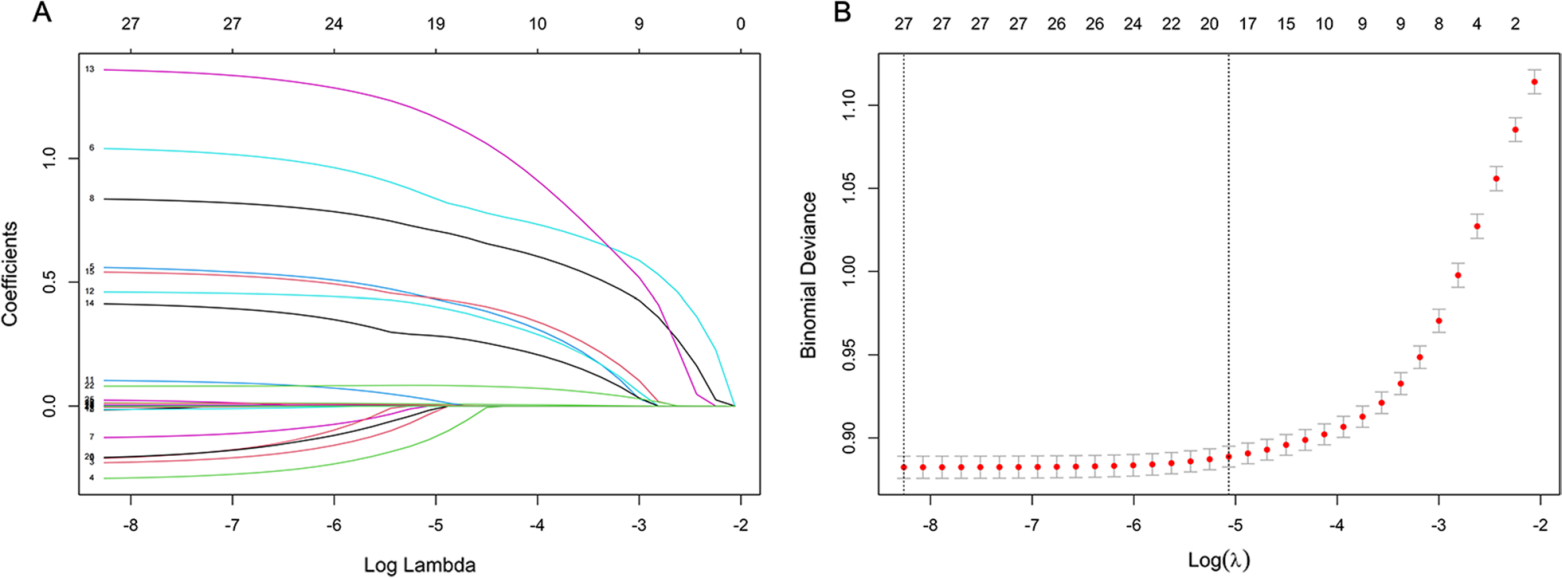
Features selection by LASSO. **A** LASSO coefficients profiles (y-axis) of the 27 features. The upper x-axis is the average numbers of predictors and the lower x-axis is the log(λ). **B** Tenfold cross-validation for tuning parameter selection in the LASSO model

**Fig. 2:**
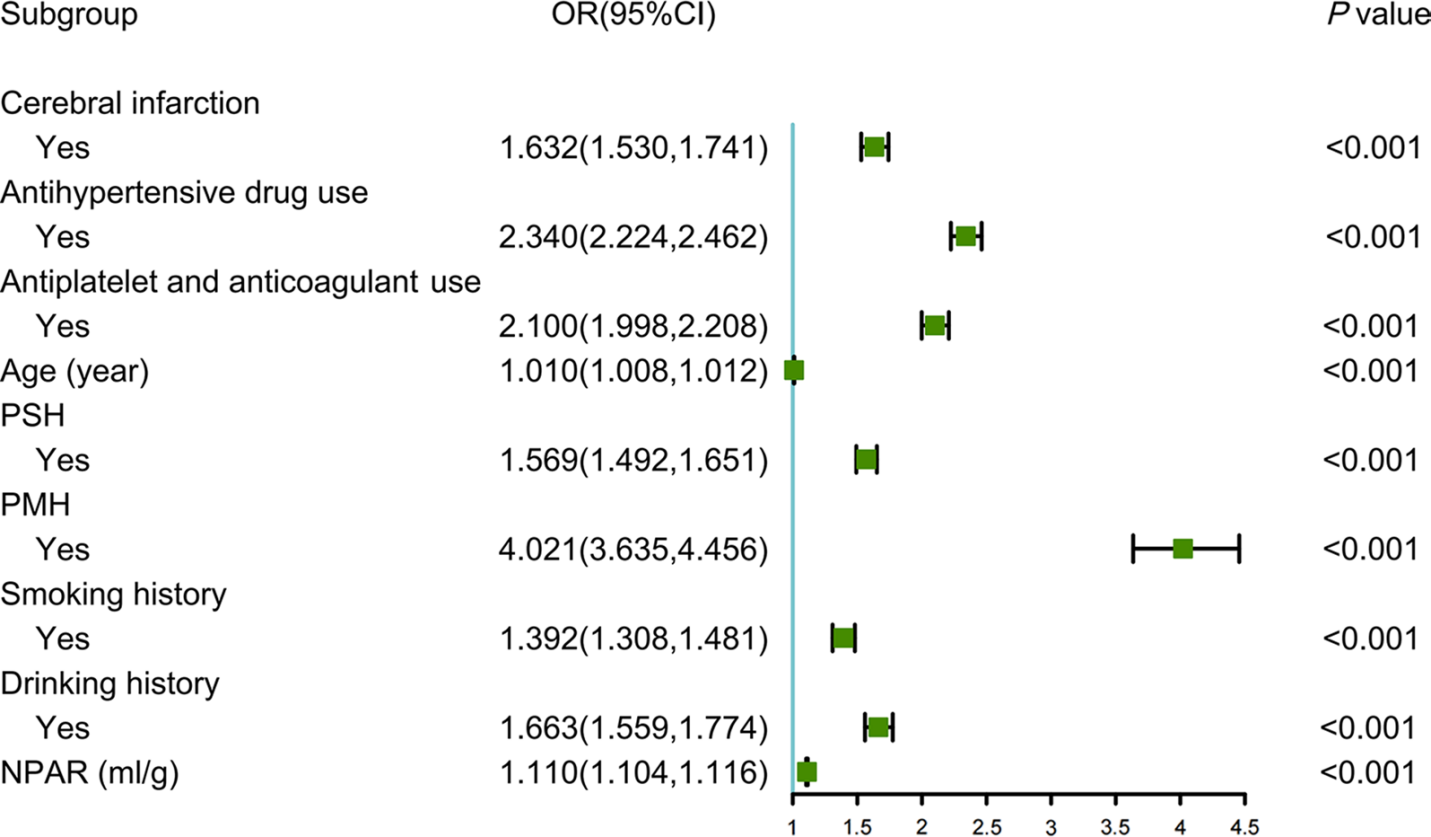
Forest plot showing the results of multivariable analysis for prolonged LOS

First, 27 variables with statistical differences in the univariate analysis were included in the least absolute shrinkage and selection operator regression. Then, 17 non-zero coefficient variables were selected at minMSE + 1SE (Fig. 1). After multivariable logistic regression, age (OR [odds ratio] = 1.010, 95% CI [confidence intervals] 1.008–1.012), cerebral infarction (OR = 1.632, 95% CI 1.530–1.741), antihypertensive drug use (OR = 2.340, 95% CI 2.224–2.462), antiplatelet and anticoagulant use (OR = 2.100, 95% CI 1.998–2.208), PSH (OR = 1.569, 95% CI 1.492–1.651), PMH (OR = 4.021, 95% CI 3.635–4.456), smoking (OR = 1.392, 95% CI 1.308–1.481), drinking (OR = 1.663, 95% CI 1.559–1.774) and NPAR (OR = 1.110, 95% CI: 1.104–1.116) were identified as final predictors of prolonged LOS (Fig. 2).

### Nomogram construction and performance

**Fig. 3:**
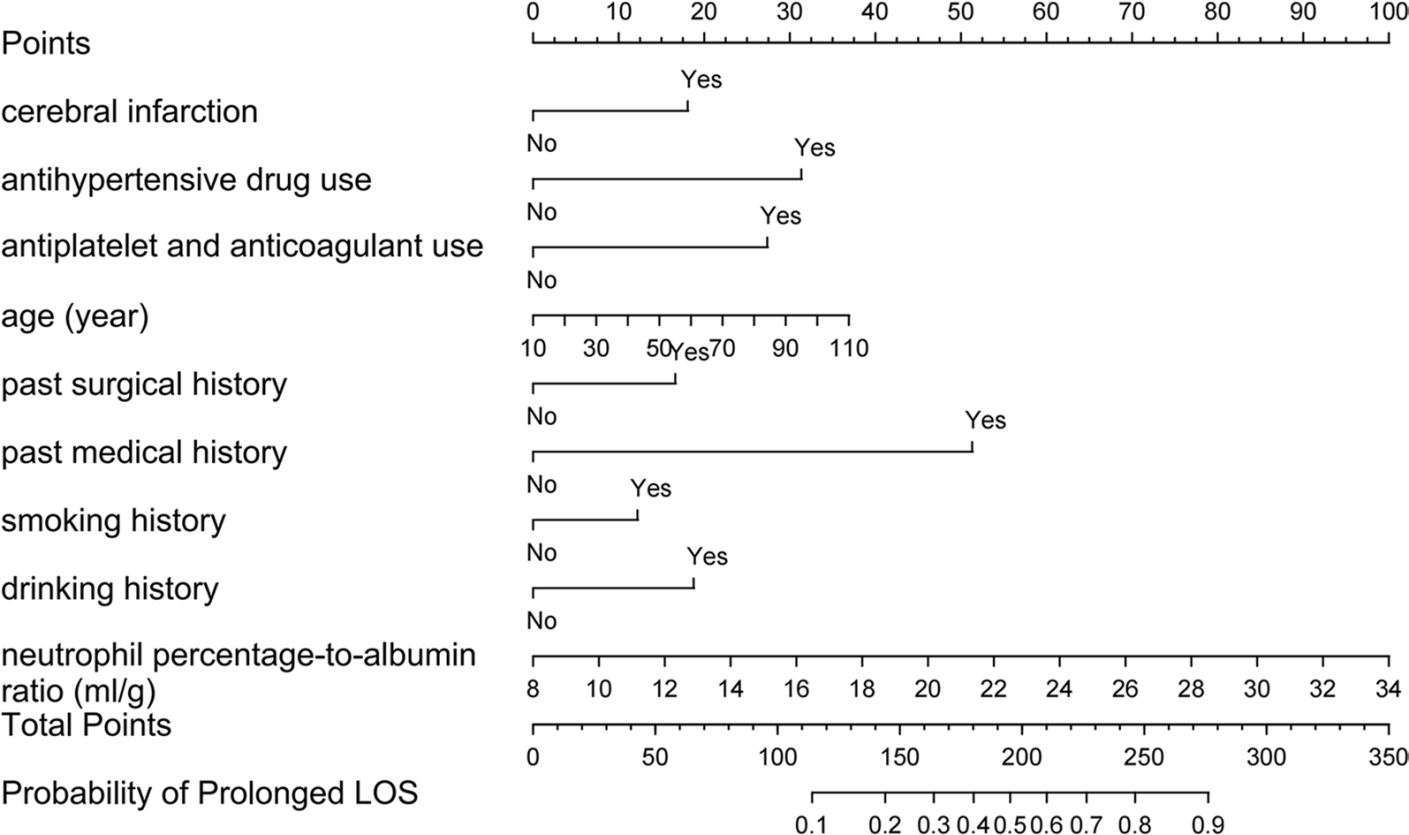
Nomogram predicting Prolonged LOS in patients with T2DM. First, a point was found for each variable of a T2DM patient on the uppermost rule; then all scores were added together and the total number of points were collected. Finally, the corresponding predicted probability of Prolonged LOS was found on the lowest rule

**Fig. 4:**
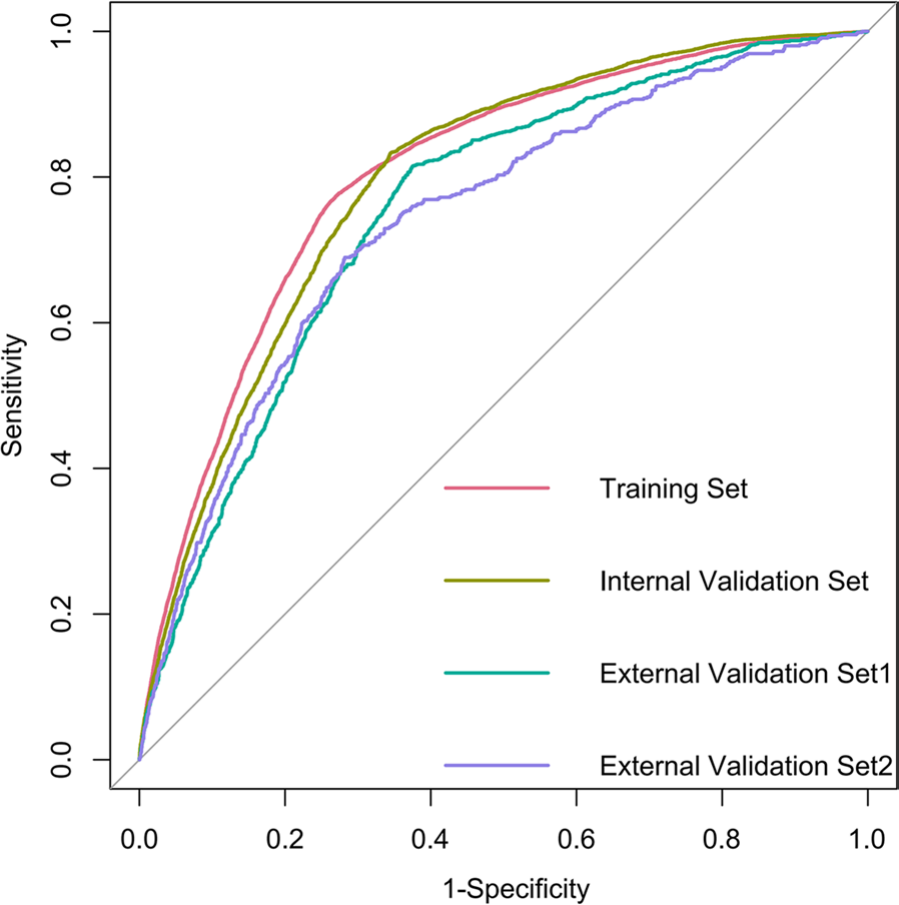
Receiver operating characteristics curves of the nomogram

**Fig. 5:**
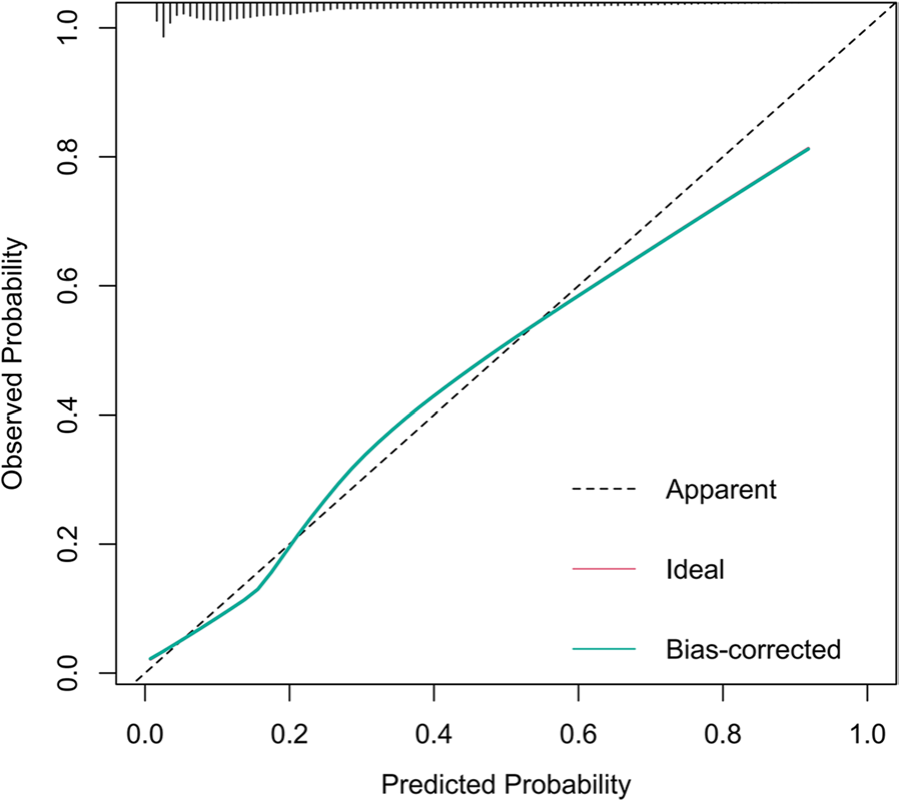
Calibration curves of the nomogram. The dotted line represents the performance of the nomogram, whereas the solid green line corrects for any bias in the nomogram. The dashed line represents the reference line where an ideal nomogram would lie

To visualize the predictive model, a nomogram was constructed, thereby providing a convenient and personalized tool to predict the probability of prolonged LOS (Fig. 3). In the training set, the area under the curve of the nomogram was 0.803 (95% CI 0.799–0.808), indicating a good discrimination (Fig. 4), and the calibration curve (bootstraps = 1000) suggested that the predicted probabilities were in good agreement with the actual probabilities (Fig. 5). The area under the curves of the internal validation set, external validation set 1 and external validation set 2 were 0.794 (95% CI 0.788–0.800), 0.754 (95% CI 0.739–0.770), and 0.743 (95% CI 0.722–0.763), respectively. Besides, the calibration curves indicated a good agreement (Additional file 1: Fig. S2–S4). More detailed performance metrics for the four models are listed in Table 3.

**Table 3:** Detailed performance metrics of the four models

Models	AUC	Sensitivity	Specificity	PPV	NPV
	(95% CI)	(95% CI)	(95% CI)	(95% CI)	(95% CI)
Training set	0.803	0.773	0.730	0.483	0.908
	0.799–0.808	0.766–0.781	0.726–0.735	0.476–0.490	0.905–0.912
Internal validation set	0.794	0.833	0.655	0.451	0.920
	0.788–0.800	0.823–0.843	0.648–0.662	0.441–0.461	0.915–0.925
External validation set1	0.754	0.815	0.625	0.270	0.952
	0.739–0.770	0.791–0.838	0.613–0.638	0.254–0.285	0.945–0.959
External validation set2	0.743	0.690	0.718	0.279	0.936
	0.722–0.763	0.654–0.725	0.704–0.732	0.258–0.301	0.927–0.944

AUC: area under the curve; PPV: positive predictive value; NPV: negative predictive value; CI: Confidence Interval

### Clinical utility of the nomogram

**Fig. 6:**
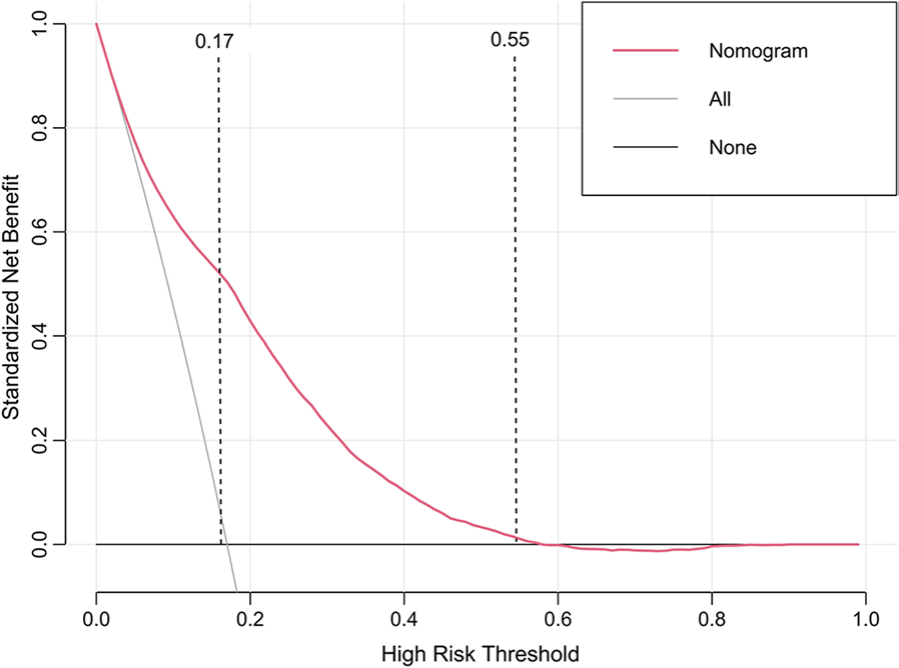
Decision curve analysis of the nomogram. Solid black line (None curve) net benefit of not investigating any people, assuming that no people would have a prolonged LOS, solid gray line (All curve) net benefit of investigating all people, assuming that all people would have a prolonged LOS

**Fig. 7:**
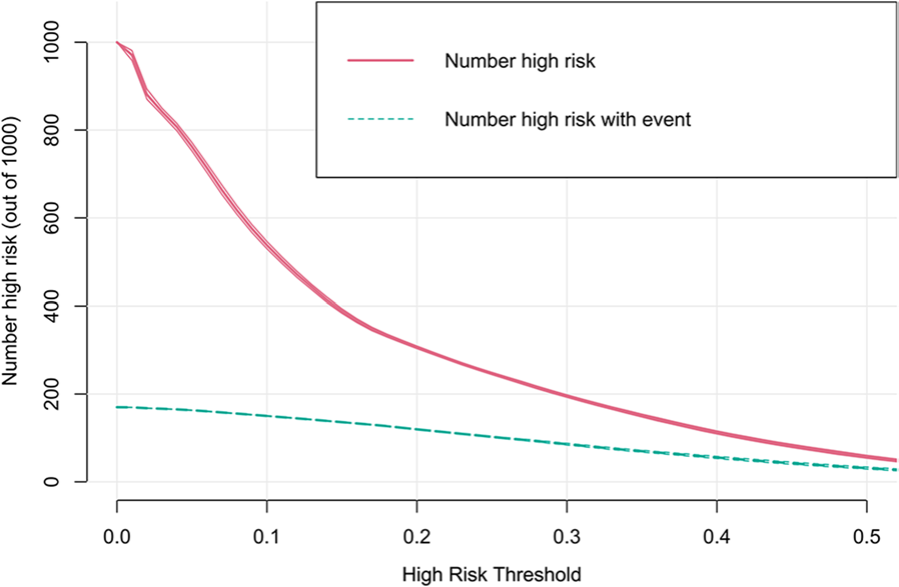
Clinical impact curve of the nomogram. The red curve shows the number of people who are classified as prolonged LOS by our model under each threshold probability; The green curve represents the number of people who are really prolonged LOS at each threshold probability

Decision curve analysis was performed to assess clinical applicability. The decision curve showed that, based on the nomogram in this study, the threshold probability of prolonged LOS in patients with T2DM was of 17–55% (Fig. 6), and application of this nomogram to predict prolonged LOS would add significantly more benefit than either the treat-all scheme or the treat-none scheme. Moreover, the clinical impact curve was further drawn according to the decision curve analysis to evaluate the clinical impact of the nomogram and thereby intuitively understand its substantive value (Fig. 7). The clinical impact curve depicted the estimated number of patients expected to reach prolonged LOS at each risk threshold and the number of patients experiencing prolonged LOS. When the risk threshold exceeded 30%, the estimated number of patients was close to the actual number of patients.

### Construction of online interface to easily access the nomogram

**Fig. 8:**
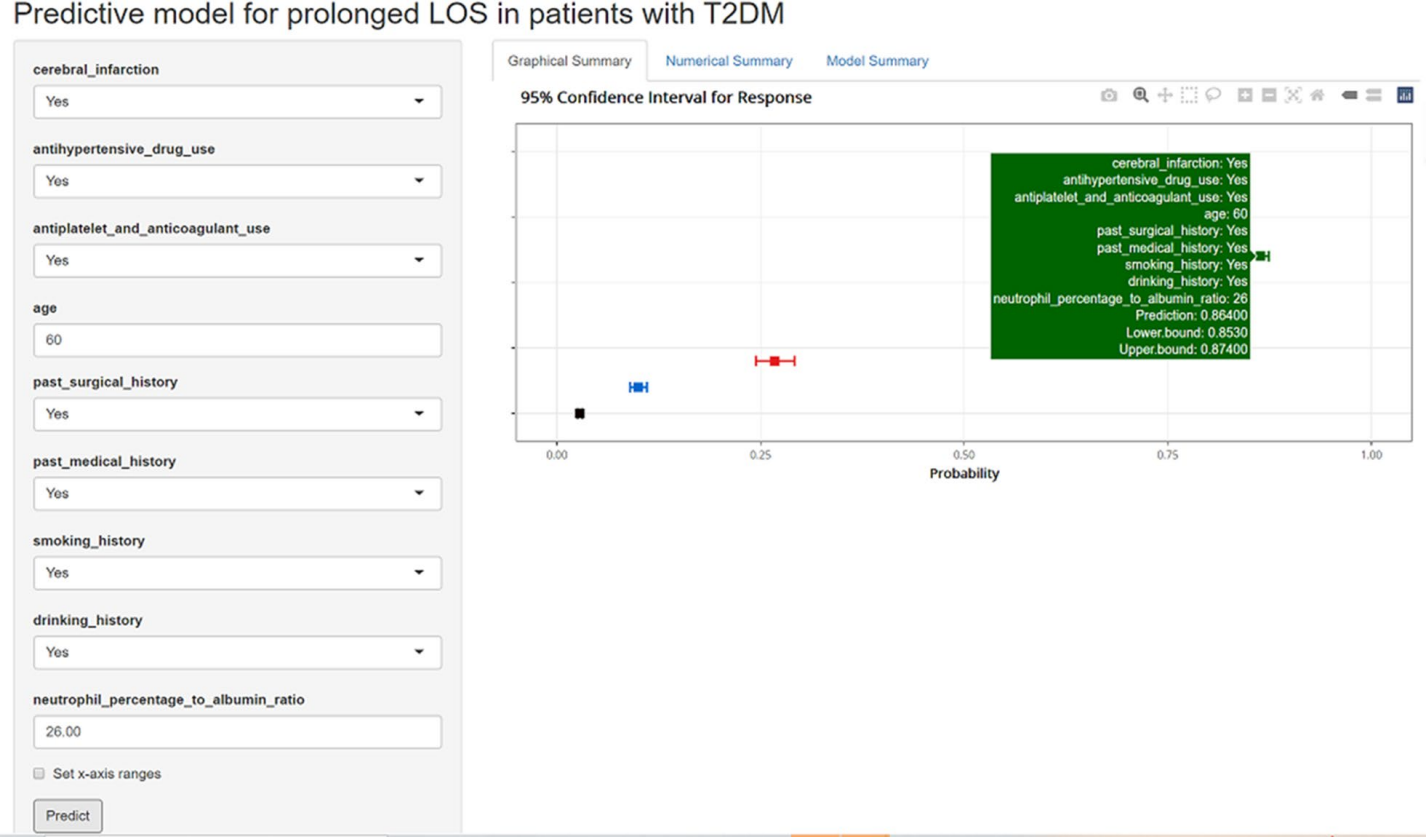
An example of nomogram to predicting prolonged LOS in patients with T2DM via a link

To facilitate the use of the nomogram by clinicians, we built an online interface (https://cytjt007.shinyapps.io/prolonged_los/) to calculate the probability of prolonged LOS. For example, when a patient has cerebral infarction, antihypertensive drug use, antiplatelet and anticoagulant use, PSH, PMH, smoking, drinking, an age of 60 years, and NPAR level of 26.00 ml/g, the probability of prolonged LOS would be 0.864 (95% CI 0.853–0.874) (Fig. 8).

## Discussion

In this study, we found that the combination of cerebral infraction, antihypertensive drug use, antiplatelet and anticoagulant use, PSH, PMH, smoking, drinking, older age, and higher NPAR levels were closely related to prolonged LOS in patients with T2DM. The combination of these variables in a prediction model formed a useful clinical tool for identifying patients with possible prolonged clinical course during hospitalization. Early identification of patients with T2DM with prolonged LOS risk can provide clinicians with the opportunity to formulate personalized treatment measures, thereby shortening the hospitalization period of these patients and reducing the disease burden of patients.

The result showed that age (OR = 1.010, 95% CI 1.008–1.012) was an independent risk factor for the prolongation of hospitalization in patients with T2DM, which was consistent with the findings of exploring the factors of prolonged LOS in other populations [32–34]. However, to a large extent, age reflects the process of aging and degenerative changes in organ function; whereas, older patients with T2DM tended to be with more diseases [35, 36], which was also confirmed in this study. The result in this study showed that cerebral infarction (OR = 1.632, 95% CI 1.530–1.741) was also an independent risk factor for prolonged LOS in patients with T2DM. Therefore, the intensity of the medical intervention received by older patients and their response, to some extent, led to the prolonged LOS.

NPAR was a recently discovered viable biomarker of systemic infection and inflammation [37]. NPAR has been proven to be a prognostic factor for pancreatic cancer, sepsis, acute ST-segment elevated myocardial infarction, and restenosis after internal carotid artery stenting [38–41]. It was well known that neutrophils were classical inflammatory cells and played an important role in mediating inflammatory response [42]. Serum albumin could exert anti-inflammatory effects by specifically inhibiting the expression of adhesion molecules [43]. Owing to its simple calculation and economic efficiency, the NPAR could be implemented even in areas with backward medical conditions [44]. More importantly, as a combination of two classical laboratory indicators, NPAR could reflect the balance between anti-inflammatory and pro-inflammatory effects in vivo to a certain extent [45]. NPAR amplified the predictive value of neutrophil percentage and serum albumin, which were often ignored by clinicians, particularly when they did not deviate significantly from the normal range. The result in this study also showed that patients T2DM with higher NPAR levels were 1.110 times more likely to be hospitalized than patients T2DM with lower NPAR levels. Therefore, attention should be paid to hospitalized patients with T2DM with high NPAR levels to avoid prolonged LOS and to improve prognosis through early active and effective treatment.

Besides, PSH, PMH, smoking and drinking have been proven to be risk factors for prolonged LOS, which was consistent with the result in this study [46–49]. Interestingly, the result also showed that antihypertensive drug use (irbesartan, furosemide, nifedipine, and others) and antiplatelet and anticoagulant use (enteric-coated aspirin tablets, dabigatran etexilate capsules, and others) correlated with prolonged LOS in patients with T2DM. It could be interpreted that the underlying conditions associated with the use of drugs correlate with prolonged LOS. However, further research is needed to confirm that the effects of certain drugs contribute to prolonged LOS.

Studies regarding the prediction models of prolonged LOS for patients with T2DM are limited. The advantage of the nomogram constructed in this study is that all predictors can be easily obtained from the patients. Moreover, to simplify the clinical application of this model, we built an operation interface on the web page, which will facilitate informed decision making regarding prophylactic treatments for prolonged LOS. However, this study has several limitations. First, this was a retrospective study and sample selection bias was inevitable. However, a multicenter and relatively large training set was used to build the models, which were further subjected to external validation. Second, some potential influencing factors were ignored because of the high percentage of missing data. The addition of these factors could improve the prediction efficiency of the model. Therefore, prospective studies with more detailed data and larger sample sizes are needed to verify or update the findings.

## Conclusion

T2DM is one of the main causes of the incidence and mortality of cardiovascular diseases, leading to an increased disease burden in many countries. This study provides us a useful clinical tool to identify possible prolonged LOS patients with T2DM. The proposed model could help clinicians formulate personalized interventions and shorten the hospitalization period, thus reducing the disease burden for patients and society.

## Supplementary Information

**Additional file 1: Figure S1.** Flow of inclusions and exclusions. **Table S1.** Information of 6 institutions in This Study. **Figure S2.** Calibration curves of the nomogram in the internal validation set. **Figure S3.** Calibration curves of the nomogram in the external validation set1. **Figure S4.** Calibration curves of the nomogram in the external validation set2.

## Data Availability

The datasets used for this study are available on request to the corresponding author.

## Code availability

An online interface (https://cytjt007.shinyapps.io/prolonged_los/) was developed to provide convenient access for users.
