# Supplementary material for: A novel model for predicting prolonged stay of patients with type-2 diabetes mellitus: a 13-year (2010–2022) multicenter retrospective case–control study
Source: J Transl Med. 2023 Feb 7;21:91. doi: 10.1186/s12967-023-03959-1 (PMC9903472; doi:10.1186/s12967-023-03959-1)
Supplement: Supplementary file 1 — Additional file 1: Figure S1. Flow of inclusions and exclusions. Table S1. Information of 6 institutions in This Study. Figure S2. Calibration curves of the nomogram in the internal validation set. Figure S3. Calibration curves of the nomogram in the external validation set1. Figure S4. Calibration curves of the nomogram in the external validation set2. [file 12967_2023_3959_MOESM1_ESM.docx]

**Additional file 1**

**Figure S1 Flow of inclusions and exclusions**

**
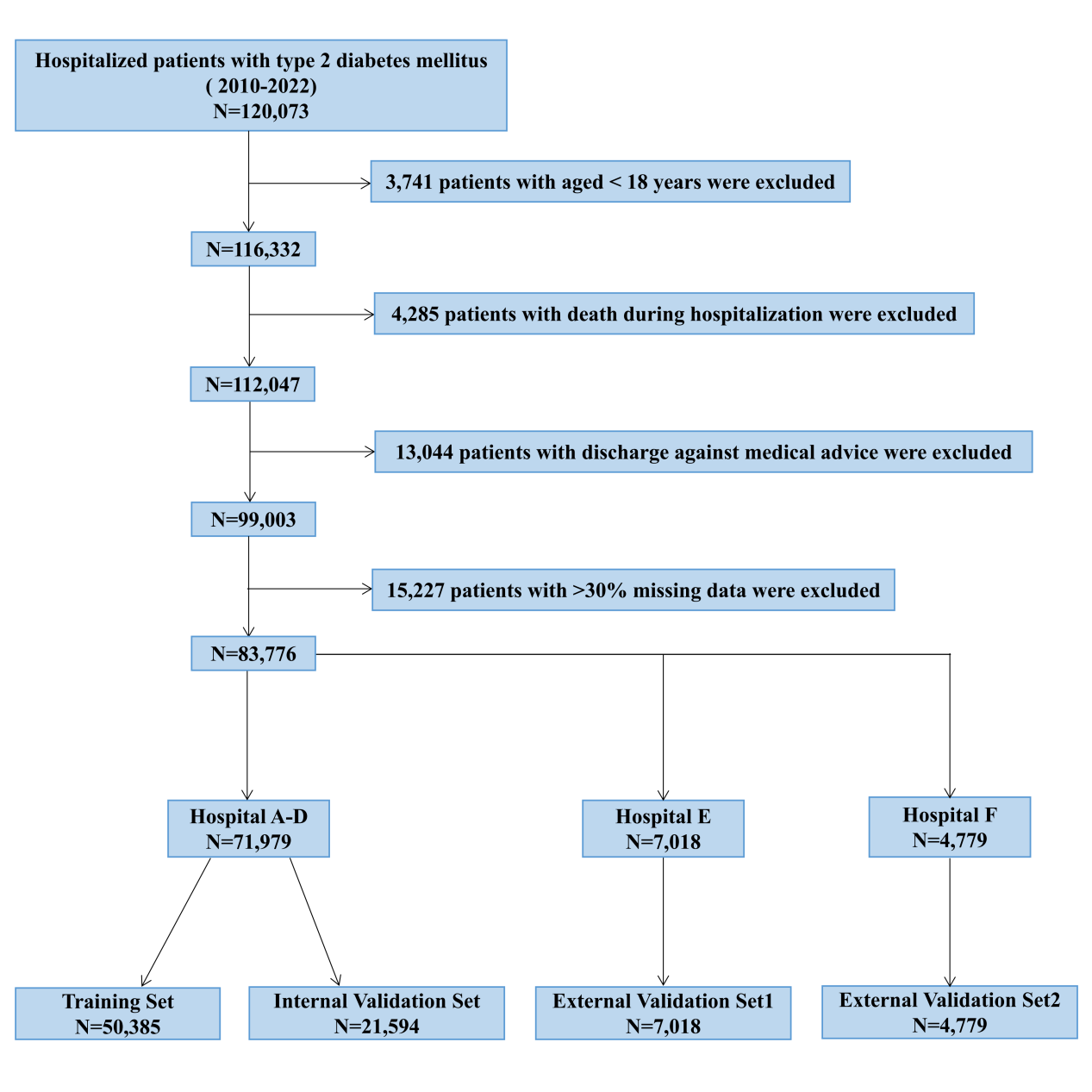
**

**Table S1 Information of 6 institutions in This Study**

| Institution | NO.of Patients Enrolled | No. of Prolonged LOS |
| --- | --- | --- |
| Yongchuan Hospital of Chongqing Medical University | 2224 | 761 |
| Second Affiliated Hospital of Chongqing Medical University | 34888 | 9632 |
| University-Town Hospital of Chongqing Medical University | 14767 | 3560 |
| Third Affiliated Hospital of Chongqing Medical University | 20100 | 3888 |
| Chongqing Southeast Hospital | 7018 | 1019 |
| People's Hospital of Tongliang District | 4779 | 654 |

**
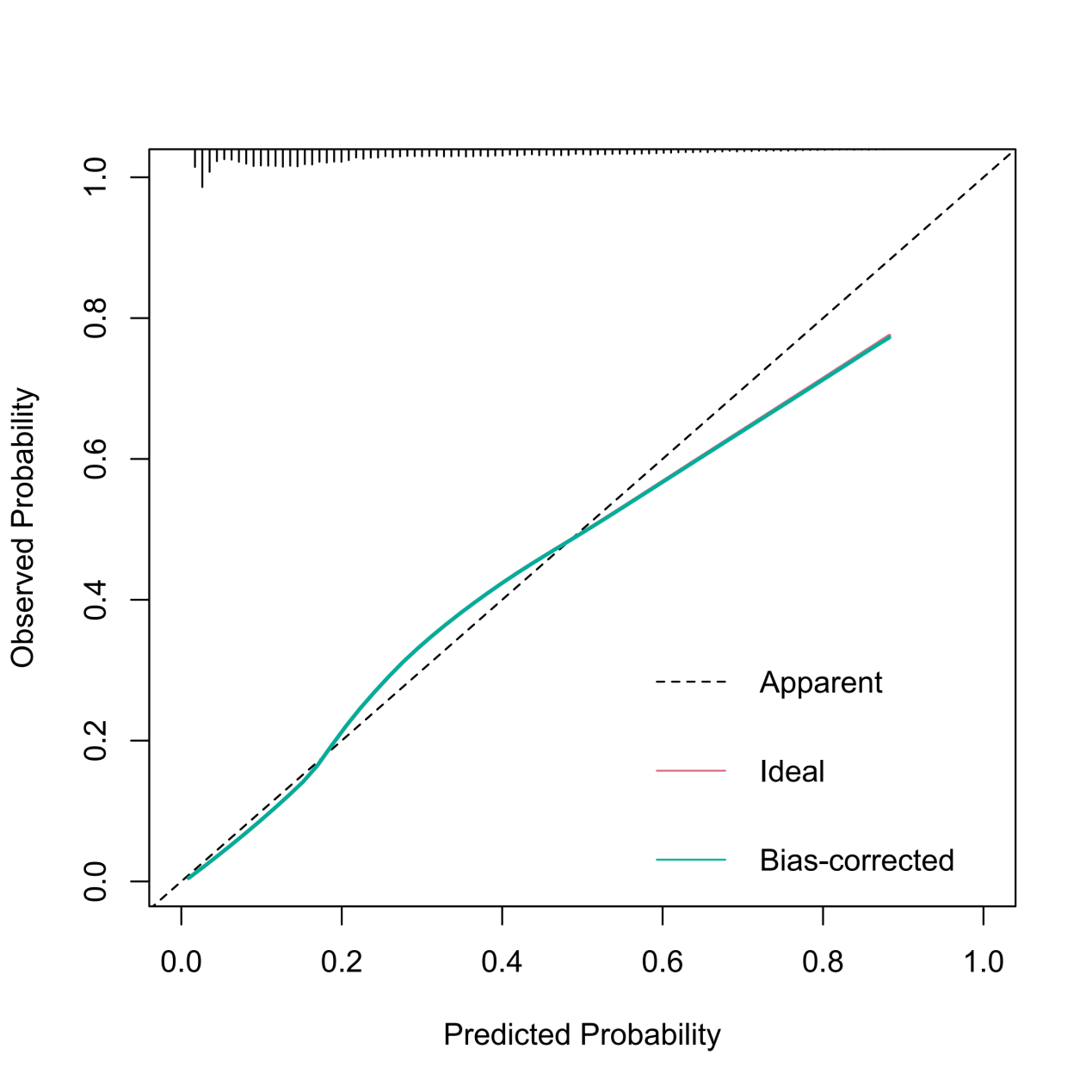
**

**Figure S2 Calibration curves of the nomogram in the internal validation set.**

**
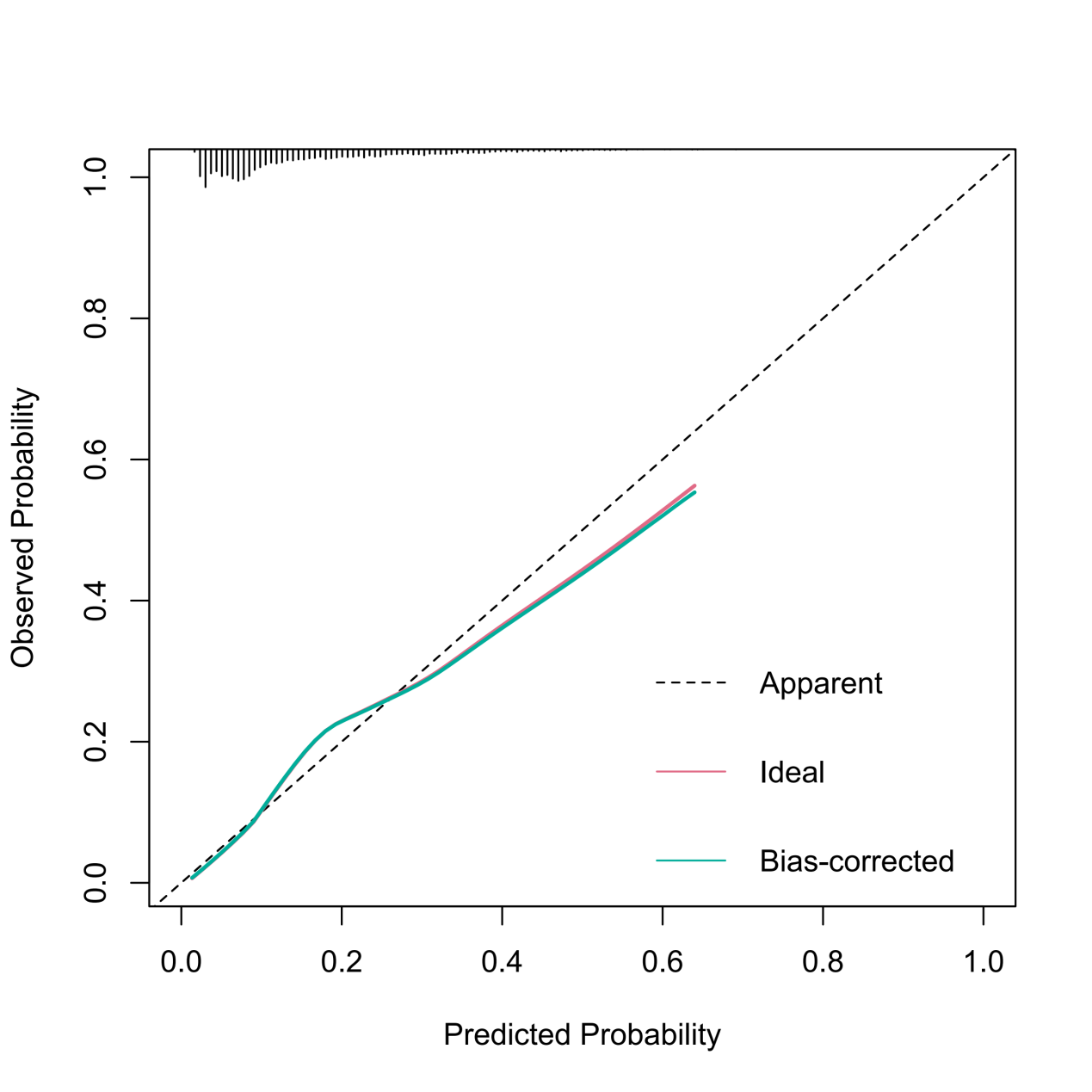
**

**Figure S3 Calibration curves of the nomogram in the external validation set1.**

**
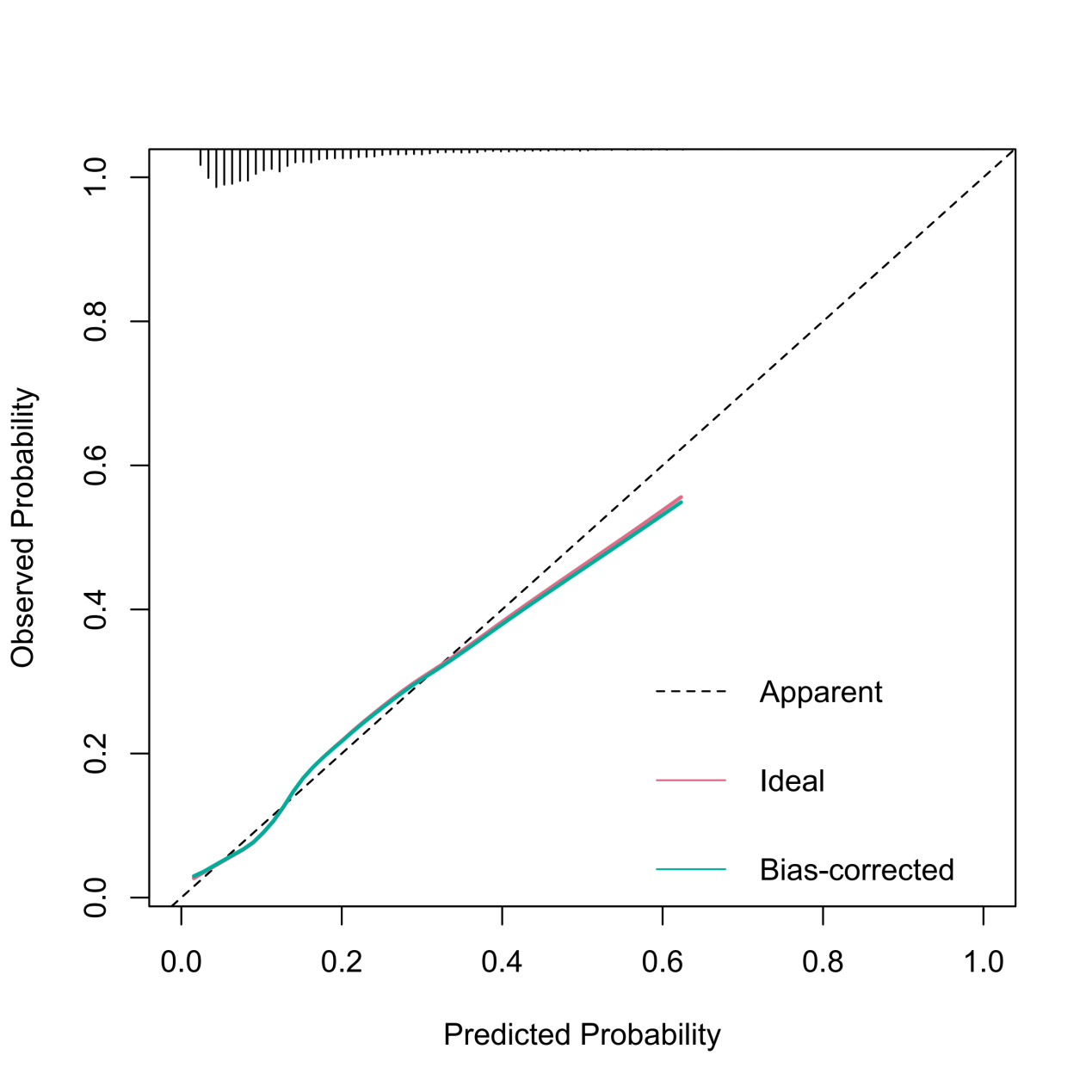
**

**Figure S4 Calibration curves of the nomogram in the external validation set2.**
